# Documented perioperative adverse events in paediatric anaesthesia in Mongolia: a first national baseline report

**DOI:** 10.3389/fped.2026.1830735

**Published:** 2026-08-19

**Authors:** Zolzaya Chinzorig, H. Reza Kahlaee, Kherlen Ponkhoon, Sergelen Sosor, Khulan Munkhtogtokh, Oyun Bayarsaikhan, Urantuya Khorolsaikhan, Khuanysh Ayatkhan, Bat-Undral Enkhbaatar, Ariunaa Purevsuren, Ariunzul Narmandakh, Ganzul Baasandorj, Justin Skowno

**Affiliations:** 1 School of Medicine, Department of Critical Care and Anaesthesiology, Mongolian National University of Medical Sciences, Ulaanbaatar, Mongolia; 2 School of Child and Adolescent Health, Faculty of Medicine and Health, The University of Sydney, Sydney, NSW, Australia; 3 Department of Anaesthesia, The Children’s Hospital at Westmead, Sydney, NSW, Australia; 4 School of Medicine, Department of Pediatrics, Mongolian National University of Medical Sciences, Ulaanbaatar, Mongolia; 5 Department of Anesthesia and Operating Block, Pediatric Hospital, National Centre for Maternal and Child Health of Mongolia, Ulaanbaatar, Mongolia; 6 Department of Anesthesia and Intensive Care, Sharav.Kh Memorial Diagnostic and Treatment Center of Umnugobi Province, Umnugovi, Mongolia; 7 Department of Anesthesia and Intensive Care, Dornod Medical Centre, Dornod, Mongolia; 8 Anesthesia and Surgical Services Department, Bayan-Ulgii Province General Hospital, Bayan-Ulgii, Mongolia; 9 Anesthesia and Surgical Services Department, Regional Diagnostic and Treatment Center in Uvurkhangai Province, Uvurkhangai, Mongolia; 10 Department of Anesthesia and Intensive Care, Magsar.Ts Memorial Bayankhongor Province General Hospital, Bayankhongor, Mongolia; 11 Department of Anesthesiology and Surgery, Central Military Hospital, Ulaanbaatar, Mongolia; 12 Department of Anesthesiology and Surgery, Mongolia-Japan Hospital of Mongolian National University of Medical Sciences, Ulaanbaatar, Mongolia

**Keywords:** anaesthesia-related event, complication, morbidity, mortality, paediatric anaesthesia

## Abstract

**Background:**

Paediatric anaesthesia safety depends on reliable data regarding perioperative adverse events. However, many low- and middle-income countries lack integrated reporting systems, making it difficult to estimate risk, identify modifiable factors, or monitor safety improvement. Mongolia has developed paediatric anaesthesia services through national training and international collaboration; however, no national system currently exists for systematic reporting of paediatric anaesthesia-related adverse events. This study aimed to provide the first national baseline description of documented perioperative adverse events in paediatric anaesthesia in Mongolia.

**Methods:**

We conducted a multicentre retrospective chart review at the main tertiary paediatric hospital and five provincial hospitals providing paediatric surgical services in Mongolia. All paediatric anaesthetic procedures performed between 1 January 2018 and 31 December 2022 were eligible. Anaesthesia charts, recovery room records, intensive care records, and other relevant perioperative documents were manually reviewed. Documented adverse events were classified as cardiovascular, respiratory, neurological, or other perioperative events. Results were summarized descriptively at the record and event levels.

**Results:**

Across 60,546 paediatric anaesthetic procedures, 640 records contained documentation of at least one perioperative adverse event. After exclusion of three records with insufficient documentation, 637 records were included in the final analysis. These records included a total of 1,159 documented perioperative adverse events, corresponding to 19.1 documented events per 1,000 procedures. Cardiovascular events were the most frequently documented category (*n* = 476, 41.1%; 7.9 per 1,000 procedures), followed by respiratory events (*n* = 283, 24.4%; 4.7 per 1,000), other perioperative events (*n* = 264, 22.8%; 4.4 per 1,000), and neurological events (*n* = 136, 11.7%; 2.2 per 1,000). The most common event subtypes were tachycardia, laryngospasm, fever, hypotension, and bradycardia. Eleven serious adverse events were documented, comprising 10 cardiac arrests and one severe neurological injury. Cardiac arrest occurred at a rate of 0.17 per 1,000 procedures. No anaesthesia-related mortality was documented in the available anaesthesia records.

**Conclusions:**

This study provides the first national baseline report on documented perioperative adverse events in paediatric anaesthesia in Mongolia. Cardiovascular and respiratory events were the most frequently documented categories, while serious adverse events were uncommon. Because this study relied on retrospective review of routine clinical records, the findings should be interpreted as documented event rates rather than definitive estimates of true incidence. These baseline data support the development of a prospective national reporting system, standardized event definitions, targeted training, and system-level safety improvements in Mongolian paediatric anaesthesia.

## Introduction

Paediatric anaesthesia remains an important area of perioperative safety because children, particularly neonates and infants, are vulnerable to rapid physiological deterioration during anaesthesia and surgery. Severe perioperative critical events continue to occur even in well-resourced settings. In the APRICOT study, a large prospective multicentre European study, severe perioperative critical events were reported in 5.2% of paediatric anaesthetic procedures, with respiratory and cardiovascular events being the most frequent categories (1). The study also suggested that the experience of the most senior anaesthesia team member was associated with a lower risk of respiratory and cardiovascular critical events, highlighting the importance of paediatric anaesthesia training, supervision, and team expertise (1). Evidence from low- and middle-income countries further supports this concern. A systematic review by Gonzalez and colleagues reported higher paediatric perioperative mortality in these settings than in many high-income countries, with airway management problems and cardiocirculatory events being major contributors to anaesthesia-related mortality (2). Together, these findings indicate that paediatric anaesthesia safety depends not only on patient vulnerability, such as young age, comorbidity, and emergency surgery, but also on system-level factors, including workforce, training, monitoring, equipment, and safety infrastructure.

System-level improvement requires reliable data on the frequency, nature, and context of perioperative adverse events. However, estimates of paediatric anaesthesia-related adverse events are highly dependent on definitions, denominators, reporting methods, case mix, and data sources (3, 4). Voluntary reporting systems can identify important safety signals but cannot reliably estimate incidence, whereas retrospective databases may misclassify events when clinical records were not designed for outcome measurement (3, 4). Prospective paediatric-specific reporting systems are therefore important for estimating incidence, identifying modifiable risks, monitoring trends, and evaluating safety interventions. Yet such systems remain limited in many low- and middle-income countries, where governance, information technology, workforce capacity and distribution, and follow-up systems may constrain large-scale data collection (3, 5, 6). Recent reviews have emphasized that the organization and delivery of safe paediatric anaesthesia in low- and middle-income countries remain affected by workforce shortages, limited paediatric-specific resources, variable access to monitoring and essential medications, and the absence of standardized safety-reporting systems (5, 6). As a result, many resource-constrained settings lack even baseline data on documented perioperative adverse events in paediatric anaesthesia care, limiting their ability to prioritize training, equipment, staffing, and quality-improvement strategies.

These gaps are directly relevant to Mongolia. Paediatric anaesthesia in Mongolia has developed through national training, international collaboration, and the expansion of paediatric surgical services. However, the country continues to face challenges common to low- and middle-income settings, including low population density, long travel distances, extreme weather, workforce constraints, and variable availability of equipment and supplies (7). At the same time, the increasing demand for paediatric anaesthesia across tertiary, provincial, and rural hospitals has not been matched by an integrated national system for systematically collecting and analysing anaesthesia-related morbidity, mortality, and perioperative adverse events. In this context, retrospective chart-review data should not be interpreted as a definitive estimate of the true national incidence of complications. Rather, such data can provide an essential starting point by describing adverse events documented in routine clinical practice. Therefore, this study aimed to provide the first national baseline description of documented perioperative adverse events in paediatric anaesthesia in Mongolia to support future surveillance, education, and system-level safety improvements.

## Methods

### Study design and setting

This was a multicentre, retrospective chart review designed to describe documented perioperative adverse events during paediatric anaesthesia care in Mongolia. The study was conducted at the National Centre for Maternal and Child Health of Mongolia, the main tertiary paediatric hospital in Ulaanbaatar, and at five provincial hospitals that provide paediatric surgical services. These hospitals were purposively selected to represent major paediatric anaesthesia services across the capital and provincial regions of Mongolia, based on paediatric surgical caseload, the availability of anaesthesia providers with paediatric experience, and the accessibility of anaesthesia-related clinical records.

The participating hospitals were the Paediatric Hospital, National Centre for Maternal and Child Health of Mongolia; Sharav.Kh Memorial Diagnostic and Treatment Centre of Umnugobi Province; Dornod Medical Centre; Bayan-Ulgii Province General Hospital; Regional Diagnostic and Treatment Centre in Uvurkhangai Province; and Magsar.Ts Memorial Bayankhongor Province General Hospital.

### Study population

All paediatric anaesthetic procedures performed at the participating hospitals between 1 January 2018 and 31 December 2022 were eligible for inclusion. The paediatric population was defined as patients from birth to less than 18 years of age. Eligible anaesthetic techniques included general anaesthesia, regional anaesthesia, procedural sedation administered by anaesthesia clinicians, or combined techniques. Cases were identified from hospital anaesthesia records and procedural logs at each institution.

The unit of analysis for the denominator was the anaesthetic procedure. Repeated anaesthetic exposures in the same patient were counted as separate procedures because each represented a distinct perioperative risk. Non-surgical procedures requiring anaesthesia care, including imaging, endoscopy, bronchoscopy, dental procedures, and procedures performed outside the operating room, were included. Procedures performed in intensive care units were excluded unless the anaesthesia team was directly involved.

For the adverse-event analysis, records were reviewed when perioperative adverse events were explicitly documented in the anaesthesia chart, recovery room record, intensive care unit record, or other relevant perioperative documentation. Records with insufficient information to confirm the event type or perioperative context were excluded from the final adverse-event analysis.

### Data sources and data collection

Data were collected by manual review of paper-based and available institutional clinical records. Source documents included anaesthesia charts, intraoperative records, post-anaesthesia care records, recovery room documentation, intensive care unit records, and other relevant hospital documents, when available.

A purpose-developed data extraction tool was created following a review of the paediatric anaesthesia safety literature and consultation with experienced Mongolian paediatric anaesthesia physicians. Data were extracted by trained anaesthesia clinicians and research assistants at each participating centre using a standardized data extraction form developed at the start of the study. A subset of records underwent independent secondary review for quality assurance, and unclear cases were reviewed by the research team and lead investigators. Discrepancies were resolved through consensus discussion.

The data extraction form included more than 80 variables covering patient characteristics, procedural characteristics, anaesthetic technique, timing of events, event category, clinical response, escalation of care, and immediate outcome. Extracted data were entered into a secure electronic database using REDCap. Data were checked for completeness and internal consistency before analysis.

### Outcomes and event classification

The primary outcome was the occurrence of chart-documented perioperative adverse events during paediatric anaesthesia care. Events were classified into prespecified categories: respiratory, cardiovascular, neurological, and other perioperative.

Respiratory events included laryngospasm, bronchospasm, apnoea, hypoventilation, aspiration, desaturation-related events, and other documented respiratory complications. Cardiovascular events included tachycardia, bradycardia, hypotension, hypertension, arrhythmia, cardiac arrest, and other documented cardiovascular instability. Neurological events included pain, shivering, neurological injury, and other documented neurological complications. Other perioperative events included fever, vomiting, hypothermia, hypoglycaemia, allergic reaction, and other documented complications.

Predefined operational definitions were used for adverse-event classification. When numerical physiological data were available, age-specific physiological thresholds based on the American Heart Association Pediatric Advanced Life Support recommendations (8) were applied, supplemented by standardized perioperative event definitions from the APRICOT study (1). Tachycardia and bradycardia were defined as heart rates above and below the age-specific reference thresholds, respectively. Hypotension was defined as systolic blood pressure below the age-specific Pediatric Advanced Life Support threshold, including systolic blood pressure <70 + 2 × age in years mmHg for children aged 1–10 years, with age-appropriate thresholds applied for neonates, infants, and older children. Fever was defined as a core body temperature ≥38.0°C during the perioperative period. When physiological values were unavailable, events were classified according to the diagnosis or event description documented by the attending anaesthesia team.

The timing of each event was categorized as preoperative, induction, maintenance, emergence or extubation, post-anaesthesia care, or intensive care/postoperative period, based on documentation in the clinical record.

Serious adverse events were defined as documented events resulting in cardiac arrest, severe neurological injury, or death. Escalation-of-care interventions and outcomes, including resuscitation interventions, airway support, vasoactive treatment, or intensive care admission, were recorded separately and were not counted as separate serious adverse events unless accompanied by cardiac arrest, severe neurological injury, or death.

Because this study was based on retrospective review of routine clinical records rather than prospective surveillance, the reported event rates represent documented adverse events and should not be interpreted as definitive estimates of the true incidence of all perioperative complications. Only adverse events explicitly documented in anaesthesia or perioperative clinical records were included in the analysis.

### Statistical analysis

The analysis was descriptive. The total number of eligible paediatric anaesthetic procedures during the study period was used as the denominator for event rate calculations. The denominator of 60,546 represented all paediatric procedures performed under anaesthesia care during the study period, including general anaesthesia, regional anaesthesia, and anaesthesia clinician-administered procedural sedation.

Categorical variables were summarized as counts and percentages. Continuous variables were summarized as mean and standard deviation or median and interquartile range, depending on distribution. Documented adverse-event rates were calculated per 1,000 paediatric anaesthetic procedures by dividing the number of documented events, or the number of records with documented events, by the total number of eligible paediatric anaesthetic procedures during the study period. For hospital-level ascertainment, rates were calculated from records initially identified as having documented perioperative adverse events before exclusion. For final analyses, records with insufficient documentation were excluded.

Record-level analyses used anaesthetic records with at least one documented adverse event as the unit of analysis. Event-level analyses used individual documented adverse events as the unit of analysis. Because more than one event could occur in the same record, the number of documented adverse events could exceed the number of records with adverse events. Percentages for event categories were calculated using the total number of documented adverse events as the denominator. Event rates were summarized overall and by clinically relevant categories, including age group, American Society of Anesthesiologists (ASA) physical status, urgency of procedure, anaesthetic period, event category, and hospital or region when available.

Because the study aimed to establish baseline descriptive data from existing records, no formal hypothesis testing was planned. Analyses were performed using Jamovi version 2.3.21. Records with insufficient information to confirm the adverse-event type or perioperative context were excluded from the final event-level analysis. Variables with partial missing data were analysed using available-case analysis without imputation, and denominators were reported where appropriate.

### Ethics

Ethical approval was obtained from the institutional ethics review committees of all participating hospitals, including the National Centre for Maternal and Child Health of Mongolia, Sharav.Kh Memorial Diagnostic and Treatment Centre of Umnugobi Province, Dornod Medical Centre, Bayan-Ulgii Province General Hospital, Regional Diagnostic and Treatment Centre in Uvurkhangai Province, and Magsar.Ts Memorial Bayankhongor Province General Hospital. Because this was a retrospective review of existing clinical records, the requirement for informed consent was formally waived in accordance with local institutional regulations. Data were de-identified before analysis.

## Results

### Study cohort and record ascertainment

**Figure 1:**
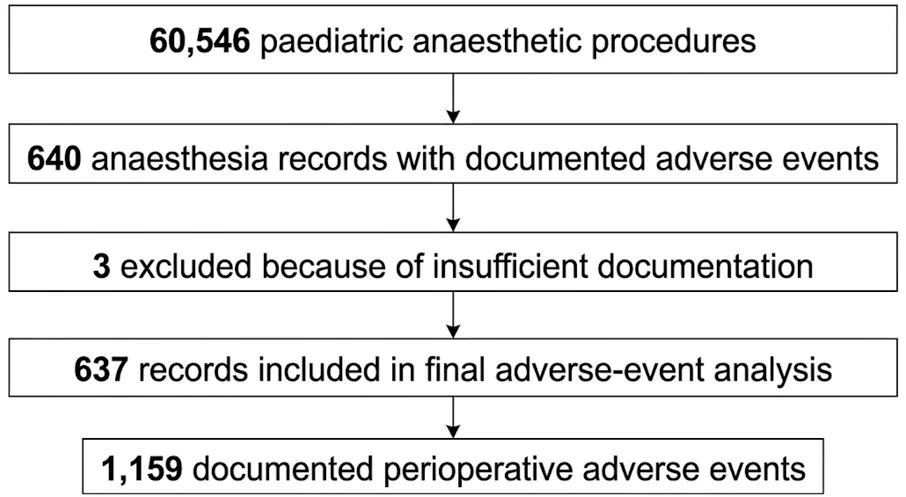
Study flow diagram.

During the study period, 60,546 paediatric anaesthetic procedures were performed across the six participating tertiary and provincial hospitals in Mongolia. Among these procedures, 640 anaesthesia records contained documentation of at least one perioperative adverse event and were initially identified for review (Table 1). Three records were excluded because the available documentation was insufficient to confirm the event type or perioperative context, leaving 637 records for the final adverse-event analysis. In total, 1,159 documented perioperative adverse events were identified among these final analysed records (Figure 1).

**Table 1:** Participating hospitals and initial ascertainment of records with documented perioperative adverse events.

Hospital	Total procedures	Records with documented events before exclusion	Rate per 1,000 procedures
NCMCH	50,923	379	7.4
Dornod Medical Centre	3,242	60	18.5
Uvurkhangai	2,225	52	23.4
Umnugobi	924	68	73.6
Bayan-Ulgii	1,657	27	16.3
Bayankhongor	1,575	54	34.3
Total	60,546	640	10.6

Rates were calculated using the number of records with documented perioperative adverse events before exclusion as the numerator and the total number of paediatric anaesthetic procedures at each hospital as the denominator. Three records were subsequently excluded due to insufficient documentation, leaving 637 records for the final adverse-event analysis. Rates are presented descriptively and should not be interpreted as direct comparisons of institutional safety because case mix, documentation practices, and reporting thresholds may have differed across hospitals.

The initial ascertainment rate of records with documented perioperative adverse events was 10.6 per 1,000 paediatric anaesthetic procedures. After exclusion of three insufficient records, the rate of records included in the final adverse-event analysis was 10.5 per 1,000 procedures. The documented event rate was 19.1 events per 1,000 procedures. The numbers of total procedures and of records initially identified as having documented perioperative adverse events varied across hospitals and regions (Table 1). These hospital-level values are presented descriptively and should not be interpreted as direct comparisons of institutional safety because case mix, documentation practices, and reporting thresholds may have differed across hospitals.

### Characteristics of records with documented adverse events

Table 2 summarizes the characteristics of the 637 records with documented adverse events. Neonates younger than 1 month accounted for 84 records (13.2%), infants aged 1 month to 1 year for 62 records (9.7%), children aged 1–3 years for 129 records (20.3%), children aged 4–8 years for 179 records (28.1%), and children older than 8 years for 183 records (28.7%). Boys accounted for 362 records (56.8%) and girls for 275 records (43.2%).

**Table 2:** Characteristics of records with documented adverse events.

Characteristic	*n*	%
Age group		
<1 month	84	13.2
1 month to 1 year	62	9.7
1–3 years	129	20.3
4–8 years	179	28.1
>8 years	183	28.7
Sex		
Boy	362	56.8
Girl	275	43.2
ASA physical status		
1	42	6.6
2	458	71.9
3	104	16.3
4	32	5.0
5	1	0.2
Urgency		
Elective	210	33.0
Emergency	398	62.5
Urgent	29	4.6
Admission status		
Outpatient/home	244	38.3
Ward	308	48.4
Intensive care unit	26	4.1
Emergency room	59	9.3
Surgical site		
Cardiac	1	0.2
Thoracic	25	3.9
Neurosurgery	2	0.3
Maxillofacial	55	8.6
Ear, nose, and throat	47	7.4
Eye	26	4.1
Upper abdomen	38	6.0
Lower abdomen	276	43.3
Perineal/anal	54	8.5
Extremity	82	12.9
Bronchoscopy	7	1.1
Esophagogastroscopy	8	1.3
Genitourinary	16	2.5
Hospital level		
Secondary or provincial	335	52.6
Tertiary	302	47.4

The values represent records with documented perioperative adverse events, not all paediatric anaesthetic procedures. Percentages were calculated using 637 analysable records as the denominator unless otherwise stated.

Most records with documented adverse events involved children classified as ASA physical status 2 or higher. ASA physical status 1 accounted for 42 records (6.6%), ASA 2 for 458 records (71.9%), ASA 3 for 104 records (16.3%), ASA 4 for 32 records (5.0%), and ASA 5 for one record (0.2%). Emergency procedures accounted for 398 records (62.5%), while 29 procedures (4.6%) were classified as unable to be delayed. Thus, urgent or emergency procedures represented approximately two-thirds of records with documented adverse events.

Lower abdominal surgery was the most common surgical category among records with documented adverse events, accounting for 276 records (43.3%). This was followed by extremity surgery (*n* = 82, 12.9%), maxillofacial surgery (*n* = 55, 8.6%), perineal or anal surgery (*n* = 54, 8.5%), and ear, nose, and throat surgery (*n* = 47, 7.4%). Secondary or provincial hospitals accounted for 335 records (52.6%), and the tertiary paediatric hospital accounted for 302 records (47.4%).

### Overall profile of documented perioperative adverse events

Table 3 summarizes the event-level profile of the 1,159 documented perioperative adverse events. Cardiovascular events were the most frequently documented category, accounting for 476 events (41.1%; 7.9 events per 1,000 procedures). Respiratory events accounted for 283 events (24.4%; 4.7 per 1,000), other perioperative events for 264 events (22.8%; 4.4 per 1,000), and neurological events for 136 events (11.7%; 2.2 per 1,000) (Table 3).

**Table 3:** Profile of documented perioperative adverse events.

Event category/subtype	*n*	% of all documented events	Documented event rate per 1,000 procedures
Panel A: Event categories			
Cardiovascular events	476	41.1	7.9
Respiratory events	283	24.4	4.7
Other perioperative events	264	22.8	4.4
Neurological events	136	11.7	2.2
Total documented events	1,159	100.0	19.1
Panel B: Leading documented event subtypes			
Tachycardia	201	17.3	3.3
Laryngospasm	187	16.1	3.1
Fever	166	14.3	2.7
Hypotension	126	10.9	2.1
Bradycardia	88	7.6	1.5
Pain	57	4.9	0.9
Shivering	50	4.3	0.8
Hypoventilation	38	3.3	0.6
Vomiting	37	3.2	0.6
Arrhythmia	30	2.6	0.5

The values represent documented perioperative adverse events, not unique patients. Panel A shows the distribution of all documented events by major event category. Panel B shows the most frequently documented event subtypes and is not intended to sum to the total number of documented events. Percentages were calculated using the total number of documented events as the denominator (*n* = 1,159). Event rates per 1,000 procedures were calculated using 60,546 paediatric anaesthetic procedures as the denominator. Because this was a retrospective chart review based on routine documentation, these values should be interpreted as documented event rates rather than true incidence estimates.

The most frequently documented individual adverse-event subtypes were tachycardia (*n* = 201, 17.3%; 3.3 per 1,000 procedures), laryngospasm (*n* = 187, 16.1%; 3.1 per 1,000), fever (*n* = 166, 14.3%; 2.7 per 1,000), hypotension (*n* = 126, 10.9%; 2.1 per 1,000), and bradycardia (*n* = 88, 7.6%; 1.5 per 1,000) (Table 3). Other commonly documented subtypes included pain, shivering, hypoventilation, vomiting, and arrhythmia. Because some records contained multiple events, these values represent event-level counts rather than unique patient counts.

Among records with documented adverse events, children aged 1–8 years accounted for nearly half of the analysed records, followed by children older than 8 years, neonates younger than 1 month, and infants aged 1–12 months. These values describe the distribution of records with documented adverse events and should not be interpreted as age-specific incidence rates, as age-specific denominators for all anaesthetic procedures were not available.

### Serious adverse events and escalation of care

Eleven serious adverse events were documented, comprising 10 cardiac arrests and one severe neurological injury (Table 4). Cardiac arrest was documented in 10 cases, corresponding to 0.17 per 1,000 procedures, or approximately 1.7 per 10,000 procedures. Nine of the 10 cardiac arrest cases occurred in neonates. Seven involved premature neonates, and eight involved children with congenital diseases requiring surgical treatment. Six cardiac arrest cases occurred during scheduled procedures in children classified as ASA physical status 3 or 4, whereas four occurred during emergency procedures in children classified as ASA physical status 4 or 5.

**Table 4:** Serious adverse events and escalation-of-care responses.

Serious event/escalation response	*n*	Rate per 1,000 procedures	Notes
Panel A: Serious adverse events			
Cardiac arrest	10	0.17	Nine cases occurred in neonates
Severe neurological injury	1	0.02	Associated with severe hypoxaemia
Documented anaesthesia-related death	0	0.00	No death documented in available records
Panel B: Escalation-of-care responses			
ICU admission after cardiovascular event	82	1.35	Responses may overlap
ICU admission after respiratory event	42	0.69	Responses may overlap
Resuscitation interventions documented for cardiovascular events	30	0.50	Event/action counts; responses may overlap
Resuscitation interventions documented for respiratory events	14	0.23	Event/action counts; responses may overlap
Unplanned ICU admission after a cardiovascular event	16	0.26	Responses may overlap
Unplanned ICU admission after a respiratory event	10	0.17	Responses may overlap

Panel A presents serious adverse events identified at the patient/event level. Panel B presents escalation-of-care responses recorded during management of perioperative adverse events. Escalation responses, including ICU admission, unplanned ICU admission, and resuscitation interventions, were recorded as event/action counts rather than mutually exclusive patient-level outcomes; therefore, overlap between respiratory and cardiovascular event categories was possible. Unplanned ICU admissions may represent a subset of ICU admissions. Resuscitation-intervention counts may include PALS-based responses for severe bradycardia, hypoxaemia, or cardiovascular instability and should not be interpreted as additional cardiac arrests. These counts were recorded as event/action counts and may overlap across event categories.

One severe neurological injury was documented. This occurred in a 13-year-old child classified as ASA physical status 2 undergoing emergency appendectomy and was associated with severe hypoxaemia during anaesthesia. No anaesthesia-related mortality was documented in the available anaesthesia records.

Escalation-of-care responses were also documented. ICU admission was recorded after 82 cardiovascular events and 42 respiratory events. Resuscitation interventions, including Pediatric Advanced Life Support (PALS)-based responses for severe bradycardia, hypoxaemia, or cardiovascular instability, were documented for 30 cardiovascular events and 14 respiratory events. Unplanned ICU admission was documented after 16 cardiovascular events and 10 respiratory events. These escalation-of-care data are presented as descriptive counts of events and actions rather than mutually exclusive patient-level outcomes. Therefore, overlap between respiratory and cardiovascular event categories was possible, and resuscitation-intervention or ICU-admission counts should not be interpreted as additional serious adverse events beyond the 10 cardiac arrests and one severe neurological injury described above.

## Discussion

This multicentre retrospective chart review provides the first national baseline description of documented perioperative adverse events in paediatric anaesthesia in Mongolia, based on major tertiary and provincial paediatric anaesthesia services. The principal finding was that cardiovascular events were the most frequently documented category, followed by respiratory, other perioperative, and neurological events. Serious adverse events were uncommon but clinically important, with 10 cardiac arrests and one severe neurological injury documented. No anaesthesia-related mortality was recorded in the available anaesthesia records. These findings should not be interpreted as definitive estimates of true national incidence. Rather, they establish a baseline of what is currently documented in routine clinical records and provide a starting point for prospective surveillance, targeted training, and system-level safety improvement.

The predominance of documented cardiovascular events differs from many large paediatric anaesthesia studies, in which respiratory events are often reported as the most common perioperative complications. For example, the APRICOT study reported severe critical events in 5.2% of paediatric anaesthetic procedures in Europe, with respiratory critical events more frequent than cardiovascular instability (1). A systematic review of paediatric anaesthesia-related mortality also identified airway management problems and cardiocirculatory events as major contributors to anaesthesia-related deaths, particularly in resource-limited settings (2). In the present study, however, cardiovascular events included relatively frequent and variably defined events such as tachycardia, hypotension, and bradycardia. Therefore, the higher proportion of cardiovascular events may reflect documentation patterns, event definitions, case mix, perioperative physiology, or local clinical thresholds for recording haemodynamic instability, rather than a true excess of cardiovascular morbidity. Direct numerical comparison with APRICOT, Wake Up Safe, or other international datasets should therefore be avoided unless definitions, denominators, severity thresholds, and data-collection methods are harmonized.

This interpretive caution is central to the value of the present study. Previous reviews have emphasized that paediatric anaesthesia adverse-event rates vary substantially depending on outcome definitions, data sources, and reporting mechanisms (3). Voluntary registries can identify important safety signals but cannot reliably estimate incidence because of selective reporting, whereas retrospective databases may misclassify events when the source records were not designed for outcome measurement (3). Similarly, de Graaff and colleagues showed that even electronically captured oxygen desaturation data require careful interpretation, as pulse oximetry artefacts can lead to overestimation of true hypoxaemia (4). Our study was based on manual review of routine paper and institutional records in a setting without an integrated national reporting system. The resulting data are therefore best understood as documented event-level frequencies, not as patient-level risk estimates or true complication rates.

Despite these limitations, the observed pattern of documented events has practical implications. Laryngospasm was the leading respiratory event, consistent with the known importance of airway events in paediatric anaesthesia. Airway management remains a major determinant of perioperative safety, particularly in neonates and infants, who have limited physiological reserve and are vulnerable to rapid desaturation. The NECTARINE study showed that perioperative critical events occur disproportionately in neonates and infants and that difficult tracheal intubation in this age group is associated with increased respiratory complications and other adverse outcomes (9–11). Cardiovascular instability, particularly tachycardia, hypotension, and bradycardia, was also frequently documented. Escalation-of-care responses, including airway support, vasoactive treatment, ICU admission, and resuscitation interventions, were recorded as event/action counts and should be interpreted separately from serious adverse events such as cardiac arrest or severe neurological injury. These findings suggest that airway management, recognition and treatment of haemodynamic instability, neonatal resuscitation, and escalation protocols should remain priority areas for education and quality improvement. The documentation of 10 cardiac arrests, nine of which occurred in neonates, is consistent with international evidence identifying neonates, infants, children with poor ASA physical status, and emergency surgery as high-risk groups for perioperative morbidity and mortality (2, 3, 9–11). The absence of documented anaesthesia-related mortality should be interpreted cautiously, as deaths may not have been consistently captured in anaesthesia-related records, and attribution to anaesthesia was not prospectively adjudicated. Prolonged preoperative fasting was also documented in records with adverse events; 282 of 637 records (44.3%) had fasting times exceeding 6 h. However, because this analysis was limited to records with documented adverse events, this finding should not be interpreted as evidence of an association between fasting duration and adverse-event risk.

These findings are also consistent with broader reports from low- and middle-income countries, where paediatric anaesthesia safety is influenced not only by patient-related factors but also by health system constraints. These include shortages of clinicians with paediatric anaesthesia training, limited access to age-appropriate monitoring and equipment, inconsistent availability of essential medications, weak postoperative follow-up systems, and the absence of standardized mechanisms for adverse-event reporting (5, 6). In this context, national reporting should not be viewed only as a research activity but also as a practical safety infrastructure that can guide training priorities, equipment planning, supervision, and quality-improvement programmes.

The Mongolian context is important when interpreting these findings. Paediatric anaesthesia in Mongolia has developed substantially through national training, international collaboration, and the expansion of paediatric surgical services. However, Mongolia continues to face challenges common to low- and middle-income settings, including low population density, long travel distances, extreme weather, workforce constraints, centralized specialist services, and variable availability of equipment and supplies (7). These system-level factors may influence not only the occurrence of adverse events but also the ability to recognize, rescue, document, and review them. Therefore, the findings of this study support strengthened supervision, clear escalation criteria, continuing education, and simulation-based training focused on common paediatric emergencies.

The main strength of this study is that it represents the first multicentre national baseline review of documented perioperative adverse events in paediatric anaesthesia in Mongolia. The study included the main tertiary paediatric hospital and five provincial hospitals, thereby capturing both central and regional paediatric anaesthesia practice. The large denominator of paediatric anaesthetic procedures provides important context for documented event rates and allows identification of commonly recorded event categories and serious events. In a country without a national paediatric anaesthesia incident-reporting system, such baseline data are an essential first step toward structured surveillance and quality improvement.

This study has several important limitations. First, because the study was retrospective, only events documented in existing clinical records could be captured. Underreporting is likely, particularly for transient, non-severe, or rapidly corrected events. Conversely, duplicate documentation of the same clinical episode may have occurred when multiple records described the same event. Second, event classification relied on routine clinical documentation rather than standardized prospective reporting, introducing the potential for misclassification and variability in reporting across hospitals, anaesthesiologists, and time periods. Third, the analysis was conducted primarily at the event level rather than the patient level; therefore, patients who experienced multiple events could contribute more than once to event counts, and the reported frequencies should not be interpreted as patient-specific incidence. Fourth, detailed denominators by age group, procedure type, provider characteristics, and other clinical variables were not available, limiting our ability to calculate subgroup-specific incidence rates or interpret differences between subgroups. Detailed perioperative information, including anaesthesia provider experience, anaesthetic techniques, medications, monitoring practices, and physiological variables, was also not consistently available, precluding detailed risk stratification and assessment of independent associations with adverse events. Fifth, mortality ascertainment was based on available anaesthesia-related records, and deaths occurring later in hospital or after transfer may not have been captured. Sixth, participating hospitals were purposively selected rather than randomly sampled. Although they included the national tertiary paediatric referral centre and major provincial referral hospitals, the findings may not be fully generalizable to smaller district hospitals, private institutions, or facilities with different case mixes and clinical practices. Finally, because this was a descriptive study, causal relationships between patient factors, provider experience, fasting time, anaesthetic management, and adverse events cannot be inferred.

The most important implication of this study is the need to move from retrospective chart review to prospective, standardized national reporting. A pragmatic Mongolian paediatric anaesthesia reporting system should use simple definitions, clear denominators, and a minimum dataset that can be completed in both tertiary and provincial hospitals. Such a system should distinguish between patient-level outcomes, event-level counts, serious adverse events, near misses, and escalation-of-care responses. Regular morbidity and mortality review, feedback to clinicians, and national aggregation of anonymized data would allow Mongolia to monitor trends, identify modifiable risks, and evaluate the effects of training and system interventions. International experience from paediatric anaesthesia reporting and quality-improvement collaboratives, such as Wake Up Safe, suggests that structured adverse-event reporting, root-cause analysis, multidisciplinary collaboration, and continuous feedback can strengthen institutional quality-improvement capacity and support safety improvements beyond simple data collection (12–14).

In conclusion, this study should be viewed not as a definitive estimate of the true incidence of paediatric anaesthesia complications in Mongolia, but rather as the first national baseline report of documented perioperative adverse events. The findings highlight frequent documentation of cardiovascular and respiratory events, clinically important serious events in neonates, and the need for better event definitions, documentation, and prospective surveillance. Establishing a national paediatric anaesthesia reporting system, combined with targeted training, supervision, equipment strengthening, and regular case review, is a practical next step toward improving paediatric anaesthesia safety in Mongolia.

## Data Availability

The original contributions presented in the study are included in the article/Supplementary Material, further inquiries can be directed to the corresponding author.
